# Identification and Expression Profile of the Chalcone Synthase (*CHS*) Gene Family in Litchi

**DOI:** 10.3390/ijms27146152

**Published:** 2026-07-09

**Authors:** Yunmei Li, Ranran Li, Jincan Xiao, Yuhao Liu, Ding Fan, Jiongzhi Xu, Wenhui Yang, Haifeng Jia, Haiji Qiu

**Affiliations:** State Key Laboratory for Conservation and Utilization of Subtropical Agro-Bioresources, College of Agriculture, Guangxi University, Nanning 530004, China; 2317391025@st.gxu.edu.cn (Y.L.); ranranli02@163.com (R.L.); xjcan718@163.com (J.X.); yuhaoliu@st.gxu.edu.cn (Y.L.); 2417391008@st.gxu.edu.cn (D.F.); xujiongzhi-2@163.com (J.X.); yangwh1223@163.com (W.Y.)

**Keywords:** *Litchi chinensis*, chalcone synthase, gene expression, cis-regulatory element

## Abstract

The chalcone synthase (*CHS*) genes play an important role in the biosynthesis of flavonoids and anthocyanins. However, research into the genomic characteristics and evolutionary patterns of the *CHS* gene family in lychee remains limited. In this study, six *LcCHS* genes were identified from the litchi genome. Most members of the LcCHS family consist of two exons and possess eight conserved motifs; furthermore, all members exhibit highly similar three-dimensional protein structures. *LcCHS* genes were closely related to homologs of Sapindaceae species and experienced gene duplication events during dicotevolution. Comparative colinearity analysis revealed conserved colinearity relationships among most *LcCHS* genes, and all members of the *LcCHS* family exhibited distinct colinearity with homologues in longan. *LcCHS* genes exhibited significant tissue-specific expression preferentially in reproductive tissues rather than vegetative tissues. Transcription analysis of ‘Jinggang Hongnuo’ litchi fruit development divided *LcCHS* genes into two negatively correlated expression modules and *LcCHS* members interacted with numerous transcription factors (e.g., flavonoid biosynthesis, hormone signaling and developmental regulation). Spatiotemporal expression profiling of *LcCHS* genes in ‘Guiwei’, ‘Feizixiao’ and ‘Ziniangxi’ litchi cultivars revealed obvious tissue specificity and developmental stage preference of these genes. Gene expression of *LcCHS* genes in ‘Guiwei’ high correlated with the flavonoid content. Furthermore, promoter cis-element analysis identified 34 types of cis elements in *LcCHS* promoters that participate in plant growth and development, hormone response and stress adaptation, implying that *LcCHS* genes may participate in litchi growth, hormone signal transduction, and stress-response processes. Comprehensive characterization of the chalcone synthase gene family could provide insights into the genetic improvement of litchi.

## 1. Introduction

Lychee (*Litchi chinensis* Sonn.) is an important fruit tree native to southern China, characterized by its Lingnan style. It belongs to the genus Litchi in the family Sapindaceae and is a subtropical evergreen fruit tree. Lychee is a typical diploid, with a somatic chromosome number of 30 (2n = 2x = 30), and its genome has been assembled into 15 pairs of chromosomes [1]. Currently, lychee production worldwide is relatively concentrated, primarily distributed across countries such as China, Vietnam, and India [2], with China having become the world’s largest producer of this fruit [3]. In the national lychee cultivation area in 2025, approximately 751.71 acres were planted [4], representing a decrease compared to the 2024 cultivation area (7.8513 million acres) [5]. As the lychee industry continues to advance variety improvement and innovation, its competitiveness has steadily increased. The rich and diverse colors of lychee fruit skin are one of the key factors determining the fruit’s appearance quality and influencing its commercial value. A rich variety of fruit colors is popular with consumers and is also an important breeding goal for breeders. Anthocyanins are important flavonoid compounds responsible for fruit color. The anthocyanin biosynthesis pathway is a branch of the plant flavonoid metabolic pathway and involves several genes in the phenylalanine pathway.

As vital secondary metabolites, flavonoids, an important class of secondary metabolites featuring a C6-C3-C6 carbon skeleton, are widely distributed throughout the plant kingdom [6]. Flavonoids exhibit a wide range of physiological activities, including protecting plants from UV-B radiation, cold stress, pathogens and herbivores, and positively regulating fruit coloration [7,8,9]. Additionally, flavonoids possess various pharmacological activities, such as antioxidant, anti-inflammatory, anti-diabetic and anti-hypertensive effects [10,11,12]. Although flavonoids exist in numerous plant species, the flavonoid biosynthetic pathway is relatively conserved across plants [13]. The biosynthesis of flavonoid originates from the phenylpropanoid pathway. Within this pathway, phenylalanine undergoes sequential catalysis by phenylalanine ammonia-lyase (PAL), cinnamate 4-hydroxylase (C4H), and 4-coumaroyl-CoA ligase (4CL) to yield 4-coumaroyl-CoA. Next, chalcone synthase (CHS) catalyzes the condensation reaction between one p-coumaroyl-CoA molecule and three malonyl-CoA molecules, producing naringenin chalcone. Chalcone isomerase (CHI) then converts naringenin chalcone into naringenin. Further downstream, a series of key enzymes—flavone synthase (FNS), isoflavone synthase (IFS), flavonoid 3-hydroxylase (F3H), dihydroflavonol 4-reductase (DFR), flavonol synthase (FLS), anthocyanin synthase (ANS), anthocyanin reductase (ANR), and leucoanthocyanidin reductase (LAR)—direct the metabolic flux toward the biosynthesis of flavones, isoflavonoids, dihydroflavonols, flavonols, anthocyanins, epicatechin, and catechin, respectively [14].

Flavonoids are typically classed into six major groups, among which anthocyanins are water-soluble flavonoid-based pigments that confer red, purple or blue coloration to leaves, flowers and fruits. In lychees, flavonoids accumulation is tightly correlated with pericarp color development and acts as a critical marker of fruit ripening [15]. CHS is the first rate-limiting enzyme in the plant flavonoid biosynthesis pathway. It catalyzes the pathway’s committed step by condensing malonyl-CoA and p-coumaroyl-CoA to generate chalcone, the first C15-skeleton flavonoid intermediate. This reaction largely determines overall metabolic flux through the flavonoid pathway, rendering CHS activity a core regulatory factor [16]. CHS belongs to the Class III polyketide synthase (PKS) superfamily, whose members share high amino acid sequence homology across plant species. Studies have shown that CHS expression is closely associated with flavonoid accumulation. For example, *GCHS4* expression in hybrid cultivars closely correlates with anthocyanin deposition [17]. Additionally, *CHS* gene expression is modulated by diverse biotic and abiotic stimuli, including phytohormones, light, and various stresses [18,19].

To date, the *CHS* gene family has been successfully identified and reported in various plants including grapevine (*Vitis vinifera*) [20], cucumber (*Cucumis sativus*) [21], eggplant (*Solanum melongena*) [22], tomato (*Solanum lycopersicum*) [23] and pepper (*Capsicum annuum*) [24]. However, systematic research on this gene family in lychee remains lacking. Genome-wide identification of *CHS* gene family was conducted based on high-quality lychee genome. A systematic analysis was performed to reveal the physicochemical properties, conserved motifs, phylogenetic relationships, promoter cis elements, and chromosomal distribution characteristics of the gene family, which might provide a theoretical basis for the genetic improvement in litchi.

## 2. Results

### 2.1. Identification, Physical and Chemical Properties of LcCHS Gene Family

**Figure 1:**
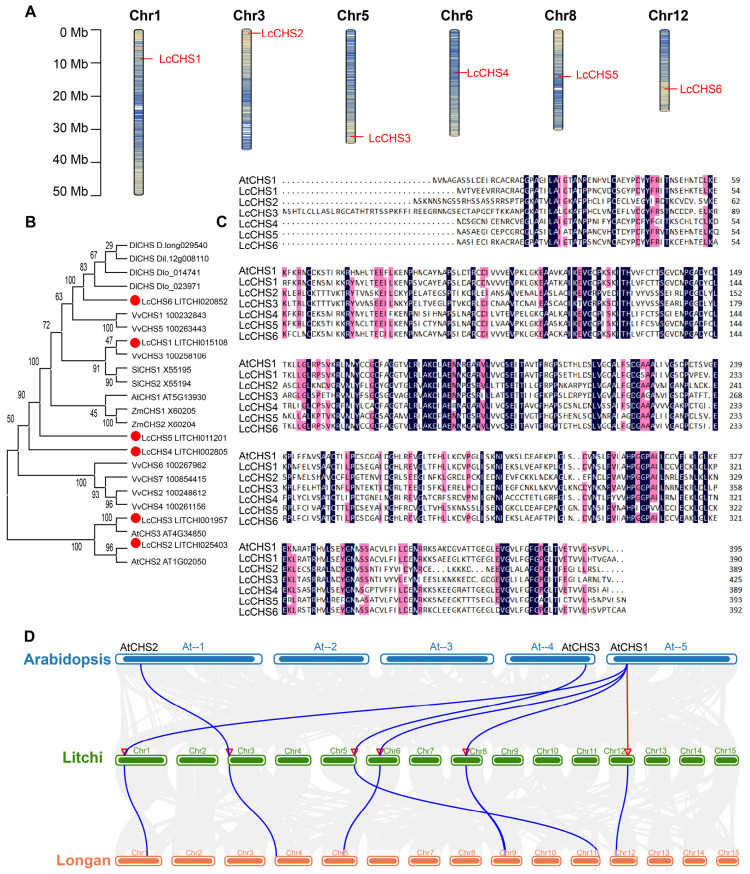
Identification and characterization of *LcCHS* family genes. (**A**) Schematic location of *LcCHS* in litchi genome. (**B**) Phylogenetic tree of the *LcCHS.* The red dots indicate six *LcCHSs* identified in this study. (**C**) Multiple sequence alignment analysis of *LcCHS*, the dark blue color indicates the conserved amino acid residues. (Dark blue indicates that the amino acid at this position is completely identical across all aligned sequences; pink indicates that at least one sequence has an amino acid residue at this position that differs from the others.) (**D**) Collinearity analysis of *CHS* genes was performed among *Arabidopsis thaliana*, *Litchi chinensis* and *Dimocarpus longan*. Red triangles indicate six *CHSs* loci in litchi. Blue lines represent syntenic relationships among Arabidopsis, litchi, and longan, while the red line highlights ortholog gene of *AtCHS1*.

Six members of the *LcCHS* family were identified in the litchi genome, designated as *LcCHS1* to *LcCHS6*. All six *LcCHS* members are distributed across six chromosomes, Chr1, Chr3, Chr5, Chr6, Chr8, and Chr12, with no two members located on the same chromosome (Figure 1A). Physicochemical property analysis revealed that the length of *LcCHS* proteins ranged from 389 to 425 amino acids, with an average length of 396 residues. *LcCHS5* encoded the longest amino acid sequence (Table S1). The relative molecular masses ranged from 42,620 to 46,861, with an average of 43,496. In addition, the isoelectric points varied from 6.13 to 7.55. and instability indices ranged between 35.36 and 47.42. Three members were categorized as stable proteins, whereas the rest were unstable with instability indices above 40. All LcCHS proteins are hydrophilic because their average hydrophilicity coefficient is negative (Table S1). Subcellular localization predictions showed that all LcCHS proteins are localized to the cytoplasm (Table S1). The secondary structure of LcCHS proteins was analyzed via SOMPA software (https://npsa.lyon.inserm.fr/cgi-bin/npsa_automat.pl?page=/NPSA/npsa_sopma.html, accessed on 22 May 2026) (Table S2). Results indicated that all LcCHS family members contain three secondary structural elements: α-helices, disordered coils, and extended chains, with no β-folds detected. The average proportion of each structural element across the full sequences, ranked from highest to lowest, was disordered coils (47.03%), α-helices (38.49%), and extended chains (14.48%). Notably, LcCHS5 showed a higher proportion of disordered coils (50.12%) compared with other family members, while its α-helix and extended chain proportions were the lowest among all paralogs (Table S2). Phylogenetic analysis was performed using 24 CHS protein sequences from seven species, including longan, tomato, Arabidopsis, and others (Figure 1B). LcCHS6 was most closely related to CHS proteins from Sapindaceae species such as *Dimocarpus longan* and its variant *Dimocarpus longan* var. JDB. In contrast, LcCHS2 and LcCHS3 clustered with Arabidopsis proteins AtCHS2 and AtCHS3, respectively. Additionally, LcCHS1 grouped with VvCHS3 of grape in the phylogenetic tree (Figure 1B). Multiple sequence alignment analysis revealed that CHS proteins in lychee contain numerous conserved amino acid residues, and these residues may serve as critical sites for their catalytic functions (Figure 1C). *Arabidopsis thaliana*, as a model plant, has been widely studied [25]. Longan and lychee belong to the same family, *Sapindaceae*, and are closely related [26]. Therefore, it is of great value to conduct synteny analysis of the *CHS* genes among them. Collinearity analysis of *CHS* genes was performed among *Arabidopsis thaliana*, *Litchi chinensis* and *Dimocarpus longan*. Extensive collinear relationships among the genomic regions harbor *CHS* genes across the three species. Specifically, *AtCHS2* and *AtCHS3* from Arabidopsis each corresponds to one *CHS* gene locus in litchi, whereas *AtCHS1* corresponded to four *CHS* gene loci in litchi. These findings indicate that the *CHS* gene family in litchi has undergone duplication events during the evolution of dicotyledonous plants (Figure 1D).

### 2.2. Characterization of LcCHS Gene Family Proteins

**Figure 2:**
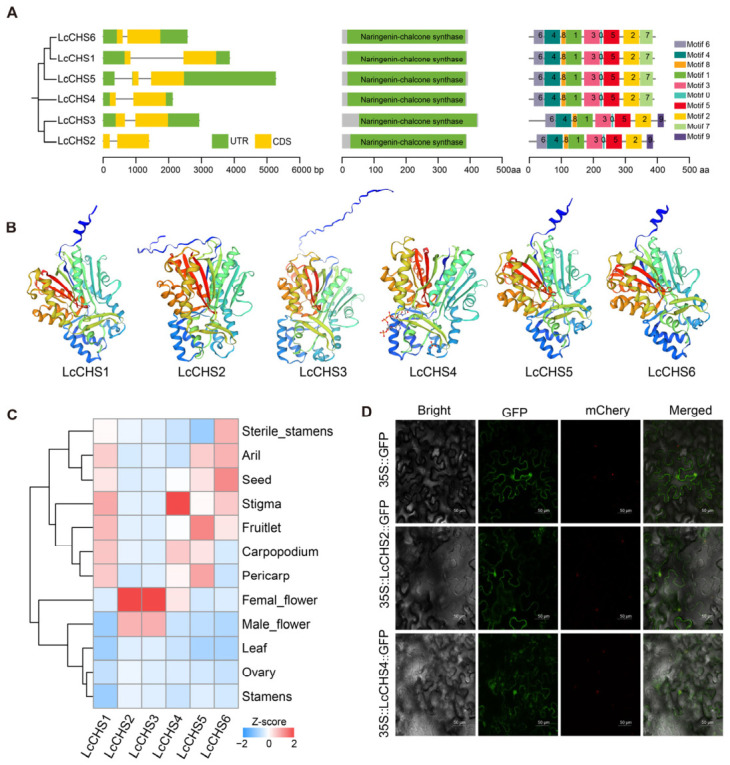
Characterization of *LcCHS* gene family proteins. (**A**) Gene structure (Green represents the Untranslated Region (UTR), and yellow represents the coding sequence (CDS)), Protein conserved domains, conserved motifs analysis of the *LcCHS* gene family. (**B**) Three-dimensional structure prediction of *LcCHS* gene family proteins. (**C**) Heatmap of the tissue-specific expression patterns of *LcCHS* genes in litchi. (**D**) Subcellular localization analysis of LcCHS2 and LcCHS4.

The gene structures of *LcCHS* family members were further analyzed. All gene family members contain two exons except *LcCHS5*, in which an intron gain event was observed. Exception for *LcCHS2*, for which no Untranslated Regions (UTRs) were annotated in the current gene model, the other members possess UTRs at both the 5′ and 3′ ends. Six LcCHS proteins contain the conserved domain of naringenin-chalcone synthase. Ten conserved motifs were identified in the *Litchi chinensis LcCHS* family. Six LcCHS family members shared eight conserved motifs, namely motif1, motif2, motif3, motif4, motif5, motif6, motif8, and motif10. Compared with the other members, LcCHS1, LcCHS4, LcCHS5, and LcCHS6 uniquely harbored conserved motif7. Similarly, LcCHS2 and LcCHS3 contained unique motif 9 (Figure 2A). Three-dimensional structure prediction of *LcCHS* gene family proteins in litchi indicated that all members shared highly similar 3D structures (Figure 2B). Expression of the LcCHS family members exhibits strict tissue specificity, with transcripts predominantly accumulating in flowers, stigmas, seeds, and young fruits, but remaining almost undetectable in leaves, stamens, and ovaries. (Figure 2C). *LcCHS1* showed constitutive but low expression across all tissues. In contrast, *LcCHS2* and *LcCHS3* were specifically and highly expressed in female flowers; *LcCHS4* reached its highest expression in the stigma, *LcCHS5* was relatively abundant in fruit tissues including fruitlets and pericarps, and *LcCHS6* was predominantly expressed in seeds and sterile stamens (Figure 2C). Subcellular localization analysis revealed that LcCHS2 and LcCHS4 were both localized in the cytoplasm (Figure 2D and Table S1).

### 2.3. Phylogenetic Tree and Conserved LcCHS Gene Blocks in the Genomes of Six Plant from Sapindaceae

**Figure 3:**
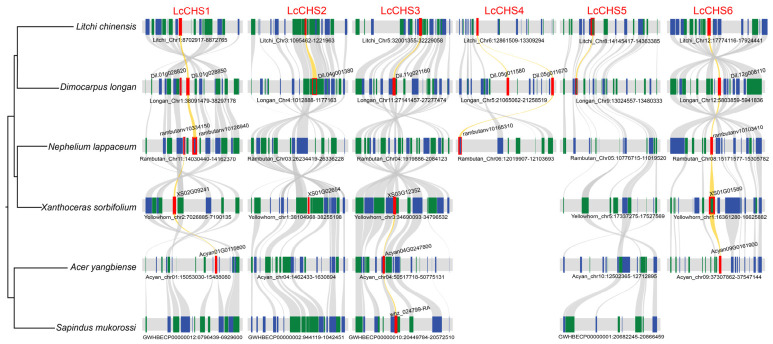
Phylogenetic relationships and conserved syntenic blocks of *CHS* genes in six *Sapindaceae* species. The phylogenetic tree on the left indicates the evolutionary relationships among *Litchi chinensis*, *Dimocarpus longan*, *Nephelium lappaceum*, *Xanthoceras sorbifolium*, *Acer yangbiense*, and *Sapindus mukorossi*. The colored vertical bars represent genes located on the corresponding chromosomes or genomic scaffolds, and the gray curves indicate conserved collinear gene pairs among species.

Collinearity analysis of *LcCHS* genes was performed across six species from Sapindaceae, including *Litchi chinensis*, *Dimocarpus longan*, *Nephelium lappaceum*, *Xanthoceras sorbifolium*, *Acer yangbiense*, and *Sapindus mukorossi*. Clear syntenic relationships were identified for all six *LcCHS* genes in closely related species. Specifically, *LcCHS1*, *LcCHS2*, *LcCHS3*, and *LcCHS6* exhibited syntenic orthologs in *D. longan*, and *X. sorbifolium*. In contrast, *LcCHS4* showed syntenic orthologs only in *D. longan* and *N. lappaceum*, with no collinear counterparts detected in *X. sorbifolium*, *A. yangbiense*, or *S. mukorossi*. All *LcCHS* genes had unambiguous syntenic orthologs in the longan genome, and a high level of collinearity was observed between their chromosomes (Figure 3).

### 2.4. Gene Expression Pattern of LcCHS During Fruit Development in Litchi

**Figure 4:**
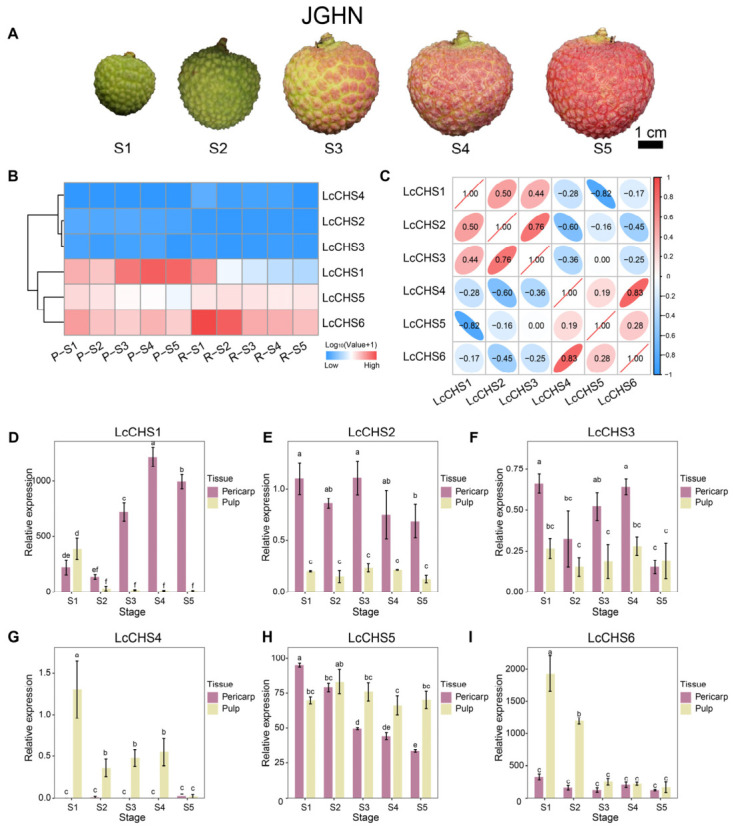
Gene expression pattern of *LcCHS* during fruit development ‘JGHN’ litchi. (**A**) The phenotype of ‘JGHN’ litchi. Schematic of sample collection from the first physiological fruit drop stage. Samples (pericarp, P; aril, R) were collected at 15-day intervals and designated S1–S5. S4, fruit coloring; S5, fruit maturity. (**B**) Heatmap of *LcCHSs* during litchi fruit development. (**C**) Correlation heatmap of *LcCHSs* during litchi fruit development. (**D**–**I**) Relative expression of six *LcCHSs* during litchi fruit development. Different letters indicate significant differences among treatments based on ANOVA followed by multiple comparisons (*p* < 0.05).

**Figure 5:**
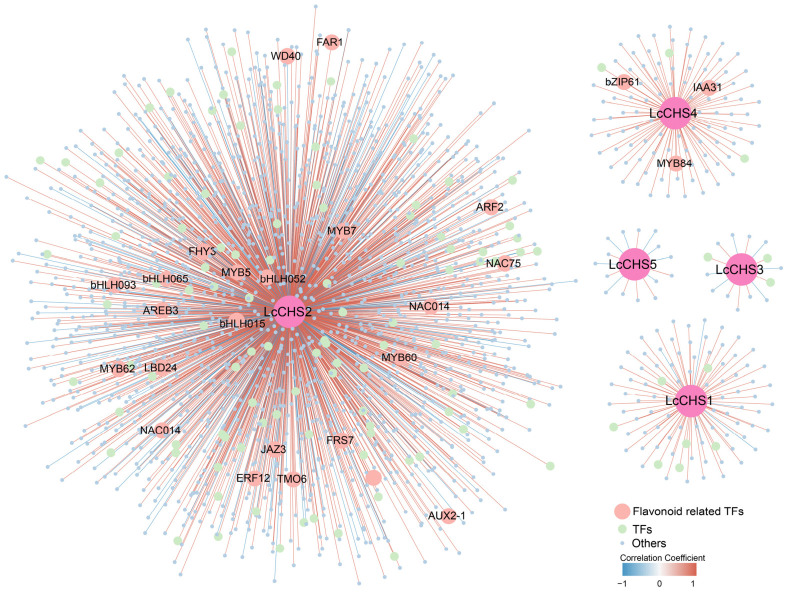
Correlation network of *LcCHS* in fruit development in litchi. Pink nodes represent flavonoid-related TFs, green nodes represent other TFs, and blue nodes represent other co-expressed genes. Red and blue edges indicate positive and negative correlations, respectively, with color intensity reflecting the correlation coefficient. Larger central pink nodes represent *LcCHS* genes.

Five fruit development stages of ‘Jinggang Hongnuo’ litchi were collected for transcriptome analysis (Figure 4A). Gene expression of *LcCHSs* was divided into two groups. *LcCHS2*, *LcCHS3*, and *LcCHS4* exhibited consistently low expression levels across all samples (pericarp and pulp). In contrast, *LcCHS1*, *LcCHS5*, and *LcCHS6* showed relatively higher expression in specific samples (Figure 4B). However, correlation analysis revealed that *LcCHS1*, *LcCHS2*, and *LcCHS3* formed a positively correlated module, especially between *LcCHS2* and *LcCHS3*. Meanwhile, *LcCHS4*, *LcCHS5*, and *LcCHS6* constituted another positively correlated module, with *LcCHS4* and *LcCHS6* exhibiting the strongest positive correlation coefficient (r = 0.83). Notably, these two modules were negatively correlated with each other, and the most significant negative correlation was observed between *LcCHS1* and *LcCHS5* (r = −0.82) (Figure 4C). *LcCHS1*, *LcCHS2*, and *LcCHS3* exhibited distinct tissue-specific expression patterns, with significantly higher transcript abundance overall in the pericarp than in the pulp, and different expression dynamics during fruit development (Figure 4D–F). The expression patterns of *LcCHS4*, *LcCHS5*, and *LcCHS6* were in stark contrast to those of *LcCHS1*-*LcCHS3*. *LcCHS4* and *LcCHS6* were mainly expressed in the pulp, with almost no expression in the pericarp. *LcCHS5*, on the other hand, was highly expressed in both the pericarp and pulp, and its expression levels in both tissues showed a synchronous decreasing trend as the fruit developed (Figure 4G–I). Interestingly, *LcCHS2* and *LcCHS4* exhibited tissue-specific expression patterns during fruit development (Figure 4D,F). A co-expression network was constructed for LcCHSs, and the results showed that *LcCHS* genes were highly correlated (|r| ≥ 0.9) with 139 transcription factors, including MYB, bHLH, and WD40 family members related to flavonoid synthesis, as well as transcription factors related to hormone signaling (ARF, JAZ, ERF) and developmental regulation (NAC, bZIP) (Table S4). The regulatory network size of different *LcCHS* members varied significantly; *LcCHS2* was associated with the largest number of transcription factors, while the regulatory networks of *LcCHS1*, *LcCHS3*, and *LcCHS5* were relatively simple (Figure 5).

### 2.5. Gene Expression Pattern of LcCHS in Multiple Experiment of Litchi Fruit

**Figure 6:**
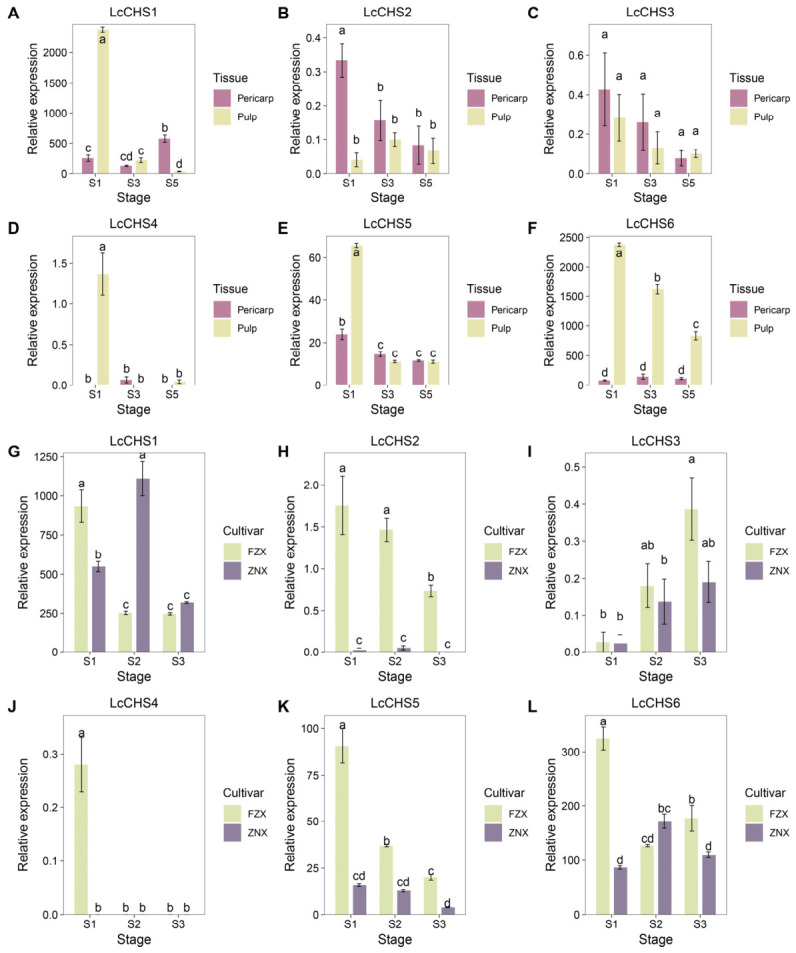
Gene expression pattern of *LcCHS* in multiple experiment of litchi fruit. (**A**–**F**) Relative expression of six *LcCHSs* during litchi fruit development of GuiWei among 41DAF (S1), 61DAF (S2) and 75DAF (S3, mature stage). (**G**–**L**) Relative expression of six *LcCHSs* during pericarp development of FZX among 17DAF (S1, small fruit), 38DAF (S2, median fruit) and 66DAF (S3, mature fruit) and ZNX litchi among 17DAF (S1, small fruit), 59DAF (S2, median fruit) and 87DAF (S3, mature fruit). Different letters indicate significant differences among treatments based on ANOVA followed by multiple comparisons (*p* < 0.05).

Analysis of the relative expression levels of *LcCHS* gene family members during the fruit development of Guiwei (GW) litchi revealed that *LcCHS* exhibited a clear tissue-biased and developmental stage-specific expression pattern. Specifically, *LcCHS1*, *LcCHS4*, *LcCHS5*, and *LcCHS6* were predominantly expressed in the pulp, while *LcCHS2* showed typical pericarp-specific expression (Figure 4A–E). *LcCHS3* showed relatively low expression in the pericarp, and its expression did not differ significantly during fruit development in the GW litchi cultivar (Figure 4C). The expression levels of most genes peaked in early fruit development (S1) and then significantly decreased with further development (Figure 6A–F). In addition, 36 flavonoids were detected across the three developmental stages. A strong correlation was observed between flavonoid contents and *LcCHS* gene expression levels. Specifically, most *LcCHS* members, including *LcCHS1*, *LcCHS4*, *LcCHS5*, and *LcCHS6*, showed a strong positive correlation with naringenin chalcone content, with Pearson correlation coefficients greater than 0.86. Naringenin chalcone is a direct product of *CHS* activity in the flavonoid biosynthesis pathway (Figure S1). Furthermore, relative quantitative analysis of *LcCHS* genes was performed on the pericarps of ‘Feizixiao’ (FZX) and ‘Ziniangxi’ (ZNX) litchi at three fruit development stages (S1, S2, and S3) [27]. The results showed significant differences in *LcCHS* gene expression patterns among varieties, and the expression trends of some genes were completely opposite in the two varieties. Overall, in FZX, the expression peaks of *LcCHS1, LcCHS2*, *LcCHS4*, *LcCHS5*, and *LcCHS6* all occurred in the early development stage (S1), and then decreased significantly with the progress of development; while in ZNX, the expression peak of *LcCHS1* occurred in the S2 stage, which was the opposite of the trend in ‘Feizixiao’. The expression levels of these genes varied between the two varieties. *LcCHS2*, *LcCHS4*, and *LcCHS5* were expressed at much higher levels in ‘Feizixiao’ than in ‘Ziniangxi’, while *LcCHS1* and *LcCHS6* showed comparable expression levels in both varieties (Figure 6G–L).

### 2.6. Cis Element Profiling in the LcCHS Gene Family

**Figure 7:**
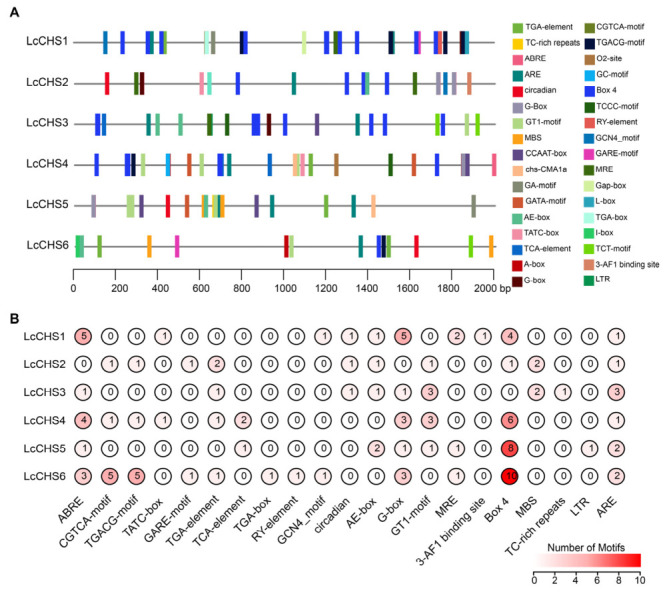
Summary of cis elements in the promoter of *LcCHS* gene. (**A**) Statistical analysis of cis elements. (**B**) Position analysis of cis elements.

Using the PlantCARE website, cis-acting elements in the promoter regions of the litchi *LcCHS* gene family were analyzed. A total of 34 types of cis elements were identified and classified into four categories, including plant hormone-responsive, stress-responsive, plant growth and development-related, and other regulatory elements (Figure 7A and Table S5). The number of cis elements varied among *LcCHS* genes, ranging from 15 to 39, suggesting differences in their transcriptional regulation.

The cis elements exhibited gene-specific distribution patterns among *LcCHS* family members (Figure 7B). ABREs were enriched in the promoters of *LcCHS1*, *LcCHS4*, and *LcCHS6*, suggesting that these genes may be involved in ABA-mediated responses. MeJA-responsive elements, including the CGTCA-motif and TGACG-motif, were especially abundant in *LcCHS6*, indicating a potential role of *LcCHS6* in jasmonate-related regulation. Auxin-responsive TGA-elements were mainly detected in *LcCHS2*, whereas salicylic acid-responsive TCA-elements were present in *LcCHS4* and *LcCHS5*. Light-responsive elements were widely distributed, with Box4 highly enriched in *LcCHS4*, *LcCHS5*, and *LcCHS6*, and G-box mainly detected in *LcCHS1*, *LcCHS4*, and *LcCHS6*. Stress-related elements also showed gene-specific patterns; ARE elements were present in all *LcCHS* genes, while the LTR element was detected only in *LcCHS5*. In addition, MBS elements were detected in *LcCHS2* and *LcCHS3*. These results indicate that individual *LcCHS* genes may be regulated by different hormonal and environmental signals, reflecting potential functional divergence among *LcCHS* family members.

## 3. Discussion

Chalcone synthase (*CHS*) belongs to a supergene family, and the copy number of *CHS* genes varies across plant species, leading to functional diversity. Research indicates that *CHS* family amino acid sequences exhibit high similarity, and their structures consist of a catalytic center formed by four residues: Cys-His-Asn-Phe. Most genes contain two exons and one intron [28,29]. This study visualized the structure of litchi *LcCHS* genes, revealing that they possess two exons and one intron, consistent with previous research.

Chalcone synthase (*CHS*) is the first rate-limiting enzyme in the plant flavonoid biosynthetic pathway. The *CHS* gene typically exists as a multi-gene family in plants and belongs to the early anthocyanin biosynthetic genes [30]. Studies in numerous species, including jasmine [31], radish [32], and lily [33], have demonstrated that *CHS* expression levels correlate closely with anthocyanin accumulation in different tissues. During the fruit development of plum, the expression of *CHS* showed a significant positive correlation with total phenolics, flavonoids, and anthocyanin content, further confirming the key role of *CHS* in regulating the accumulation of phenolic compounds in fruits [34]. The correlation network between *LcCHS* gene expression and flavonoid metabolites showed that all six *LcCHS* genes were associated with multiple flavonoid compounds, suggesting that the *LcCHS* family may participate extensively in flavonoid metabolic regulation during fruit development. Notably, *LcCHS2* and *LcCHS4* were closely correlated with naringenin chalcone, kaempferol- and quercetin-derived compounds, epicatechin glucoside and other flavonoid metabolites, indicating that they may be key candidate genes involved in flavonoid accumulation, although further functional validation is still required (Figure S1).

The expression of the *CHS* gene is closely related to organ morphogenesis and functional differentiation. Due to variations in expression patterns, *CHS* exhibits tissue specificity. In ginseng (*Panax ginseng*), transcript accumulation of the *CHS* gene is primarily detected in leaves [35]. Similarly, *CHS1* expression in mango (*Mangifera indica*) is notably higher in leaves and floral tissues compared to other organs [36]. This study indicated that the *LcCHS1* and *LcCHS2* genes are expressed in all parts of *Litchi chinensis* ‘Fei Zi Xiao’, while *LcCHS5* is only minimally expressed in male and female flowers and exclusively in flowers (Figure 2C). These tissue-specific expression patterns may be largely associated with their specific functions. This is generally consistent with previous reports, although certain differences were observed, suggesting that *CHS* gene expression levels vary among tissues across different species. Moreover, throughout the five developmental stages of the *Litchi chinensis* ‘Jinggang Hongnuo’ fruit, low expression of *LcCHS3* was detected in either the pericarp nor aril (Figure 4A,E). This finding shares the same trend with previous observations in which only four *LcCHS* genes were expressed during the fruit development of the ‘Fei Zi Xiao’ variety [37], suggesting that *LcCHS3* either lacks functional significance or plays a negligible role in the fruit development of this cultivar. This speculation is further supported by related studies in sunflower and carnation (*Dianthus chinensis*). Among the 12 *CHS* genes identified in sunflower, the expression patterns of *HaCHS1*, *HaCHS7*, *HaCHS8*, and *HaCHS11* in purple-red sunflower petals were highly consistent with the trend of anthocyanin accumulation, gradually increasing as anthocyanin content rose and petal coloration deepened [38]. Similarly, the *DchCHS1* overexpression (OE) line of *Impatiens uliginosa* exhibited darker flower color and significantly higher anthocyanin content; qRT-PCR analysis confirmed that no significant difference was detected in *DchCHS3* expression levels between the *DchCHS1-OE* and *DchCHS3-OE* lines [39]. Based on the above cross-species evidence and the relative expression levels of *CHS* genes in litchi, we propose that *LcCHS1* and *LcCHS6* are the most promising candidate genes involved in regulating fruit color accumulation. The subcellular localization analysis showed that LcCHS proteins were mainly localized in the cytoplasm, which is consistent with the reported localization of CHS enzymes in other plant species. CHS catalyzes the first committed step of flavonoid biosynthesis by converting p-coumaroyl-CoA and malonyl-CoA into naringenin chalcone, and this reaction generally occurs in the cytoplasm (Figure 2D).

Cis elements in the promoter may serve as transcription factors binding sites, thereby modulating gene transcription. Cis-element analysis revealed that the promoters of lychee *CHS* genes contain regulatory elements associated with hormone, abiotic stress, and growth/development responses, consistent with previous findings [40]. Abiotic stresses (high salinity, low temperature, drought) and hormones can induce *CHS* gene expression, with cis upstream of the promoter exerting a crucial influence on expression levels [41]. Numerous cis elements involved in growth, development, and responses to light and stress have been identified in the promoter region of the lychee *LcCHS* gene. Studies indicate that plant hormones (such as SA, ABA, and MeJA) can induce *CHS* expression and enhance *CHS* activity. ABA response elements (ABRE) and MeJA response elements (TGACG and CGTCA motifs) were identified within the *DcCHS* promoter region, indicating that *LcCHS* gene expression can also be induced and its activity enhanced by exogenous ABA or MeJA application [42,43,44]. Exogenous ethylene treatment significantly up-regulated the expression of key anthocyanin biosynthetic structural genes, including *VvCHS*, and increased the content of multiple individual anthocyanins in grape berries. This finding suggests that ethylene may also participate in regulating *CHS* expression and anthocyanin biosynthesis in non-climacteric fruits, providing new insights into hormonal regulation of *LcCHS* genes in litchi [45].

This study identified members of the lychee *CHS* gene family and analyzed their physicochemical properties, phylogenetic trees, chromosomal localization, synteny, and cis elements, revealing the diversity characteristics of this gene family. These findings will provide a reference basis for future studies on the functions of the lychee *CHS* gene family.

## 4. Materials and Methods

### 4.1. Screening and Identification of LcCHS Gene Family

Seven grape and four *Arabidopsis* CHS protein sequences were downloaded from the KEGG [46] and TAIR [47] databases, respectively, as reference sequences. Homologous sequences were obtained by alignment with the genome of the genus *Litchi* (Sapindaceae database) using the BLASTp (v2.14.1) program (identity ≥ 40%). The integrity of conserved domains of the obtained protein sequences was assessed using NCBI-CD-Search website tools [48]. Meanwhile, genes containing specific functional domains (BH0617, naringenin-chalcone synthase) were selected. The genes were named *LcCHS1* to *LcCHS6* according to their chromosomal order.

### 4.2. Physicochemical Properties, Subcellular Localisation Prediction and Protein Structure Analysis of Members of LcCHS Family

The physicochemical properties of *LcCHS* genes, including amino acid length, relative molecular mass, instability coefficient, and fat coefficient, were analyzed using the ExPASy online software (https://web.expasy.org/protparam/, accessed on 8 September 2025) [49]. Prediction for subcellular localization of the *LcCHS* genes was performed using the WoLF PSORT (https://wolfpsort.hgc.jp/, accessed on 8 September 2025) [50] and CELLO (v.2.5) online tools [51]. The secondary structure of LcCHS1-LcCHS6 proteins (including proportions of α-helix, β-sheet, disordered regions, and extended chains) was analyzed using the SOPMA online tool (https://npsa.lyon.inserm.fr/cgi-bin/npsa_automat.pl?page=/NPSA/npsa_sopma.html, accessed on 8 September 2025) [52].

### 4.3. Chromosomal Localisation and Collinearity Analysis of Members of LcCHS Family

To obtain the chromosomal location information of the *LcCHS1-LcCHS6* genes, the litchi genome annotation file was used, and TBtools (v2.420) [53] was employed to map these genes onto the chromosomes. Download *Litchi* and *Longan* genome data files from the *Sapindaceae* database [54]. Download *Arabidopsis* genome data files from the National Genome Data Center (NGDC) database [55]. Use TBtools to visualize the collinearity relationships among *CHS* family members in lychee, longan, and Arabidopsis, and present the results visually.

### 4.4. Construction of a Phylogenetic Tree of the LcCHS Family Members

Related protein or nucleotide sequences were obtained from NCBI, KEGG, or Sapindaceae databases, including those of tomato (*Solanum lycopersicum*) (NCBI: X55194.1), Arabidopsis (*Arabidopsis thaliana*) (Tair: *AT5G13930*, *AT1G02051*, *AT4G00040*, *AT4G34850*), grape (*Vitis vinifera*) (KEGG: *100232843*, *100248612*, *100258106*, *100261156*, *100263443*, *100267962*, *100854415*), maize (*Zea mays*) (NCBI: *X60204.1*, *X60205.1*), *Dimocarpus longan* ‘Hong He Zi’ (Sapindaceae Database: *Dlo_014741.1*, *Dlo_023971.1*), *Dimocarpus longan* ‘Shi Xia’ (Sapindaceae Database: *Dil.12g008110.1.t1*), and *Dimocarpus longan* ‘Ji Dan Ban’ (Sapindaceae Database: *D.long029540.01*). These sequences were imported into MEGA 7 software [56] for multiple sequence alignment. A phylogenetic tree was constructed using the Neighbor-Joining method with 1000 bootstrap replications, and all other parameters were set to the default values. The multiple sequence alignment and visualization of LcCHS proteins were performed using DNAMAN software (v6.03).

### 4.5. Conservative Motifs and Gene Structure of LcCHS Family Members

Using the MEME online platform [57], we analyzed the conserved motifs of lychee chinensis LcCHS family members (with motif count set to 10 and other parameters at default values), obtained the conserved motif information, and visualized it. The gene structure shower module in the TBtools was employed to visualize the gene structures, conserved domains and conserved motifs according to genome annotation file.

### 4.6. Three-Dimensional Structure Prediction and Spatiotemporal Analysis of LcCHSs

Three-dimensional structures of LcCHS1–LcCHS6 proteins were predicted using an online protein structure prediction server (SWISS-MODEL). The amino acid sequences of the six LcCHS proteins were submitted individually to the server with default parameters. Expression data of CHS genes in different tissues were downloaded from Sapindaceae Database (www.sapindaceae.com, accessed on 1 March 2026) based on their gene IDs. The expression values were normalized and transformed using log_2_(value + 1). A heatmap function of R program (v4.4.3) was then generated to visualize the expression patterns of CHS genes across different tissues.

### 4.7. Phylogeny and Synteny of CHS Genes in Six Sapindaceae Species

Synteny analysis of *LcCHS* genes was performed using the Synteny Analysis module in the Sapindaceae Database (www.sapindaceae.com, accessed on 1 March 2026). The gene IDs of the identified *LcCHS* members were individually submitted to the Synteny Search function to obtain the collinear relationships of homologous genes among six Sapindaceae species with available reference genomes. The phylogenetic relationships among these six species were extracted using the Sapindaceae Phylogeny function. Finally, the synteny and phylogenetic information were integrated and visualized using Adobe Illustrator (v27.00).

### 4.8. Download and Analysis of In-House and Public Transcriptome Data and Flavonoids Metabolites

In-house transcriptome data of the pericarp and aril of the litchi cultivar ‘Jinggang Hongnuo’ at five developmental stages, from 35 days after flowering to the mature stage, were obtained from our laboratory database. In addition, public transcriptome datasets were downloaded from the NCBI database, including pericarp and aril RNA-seq data of ‘Guiwei’ litchi at 47, 61, and 75 days after flowering under BioProject accession number PRJNA904256 [58] and pericarp transcriptome data of ‘Feizixiao’ and ‘Ziniangxi’ litchi under BioProject accession number PRJNA1117045 [27].

After downloading, raw RNA-seq reads were filtered using fastp (v0.23.4) to remove low-quality reads and adapter sequences. The clean reads were then aligned to the ‘Feizixiao’ reference genome using HISAT2 (v2.2.3). Gene expression levels were quantified using featureCounts (v2.0.6), and the read counts were normalized to TPM values using R (v4.4.3). Pearson correlation analysis among samples was performed using the cor (v4.4.3) function in R. Heatmaps were generated using the pheatmap (v1.0.13) package, while bar plots were drawn using ggplot2 (v4.0.1). Multiple comparisons among different samples or developmental stages were performed using Tukey’s test. Correlation networks were visualized using Cytoscape (v3.10.4).

Flavonoid metabolites were extracted from previous research coupled with RNA-seq of ‘Guiwei’ litchi [58]. The Pearson correlation analysis between gene expression of *LcCHS* and the content of flavonoid was performed using the cor function in R. The genes-flavonoid pairs with Pearson correlation coefficients greater than 0.6 or lower than −0.6 were used for correlation networks, visualized using Cytoscape (v3.10.4).

### 4.9. Gene Cloning of LcCHS2 and LcCHS4

Based on the coding sequence (CDS) of the *LcCHS2/4* gene, specific cloning primers LcCHS2-F and LcCHS2-R were designed using SnapGene (v6.0.2) software. Gene was cloned using *Litchi chinensis* JingGang HongNuo cDNA, yielding the complete coding region of the gene (excluding the stop codon). After recovery and purification of the PCR products, they were ligated with Vazyme 5 × TA/Blunt-Zero Cloning Mix (Catalog No: C601). Positive clones were screened via PCR and sent to Nanning Biotech for sequencing verification. The correct positive clones identified by sequencing are used to extract the recombinant plasmid, designated TA/Blunt-LcCHS2/4, for subsequent experimentation.

### 4.10. Construction of LcCHS2 and LcCHS4 Overexpression Vectors

Specific primers were designed using SnapGene (v6.0.2). The forward primer p1302-CHS2/4-F incorporates an NcoI restriction site, and the reverse primer p1302- CHS2/4-R incorporates a SpeI restriction site (primer sequences are shown in Table S3). Using the TA/Blunt-LcCHS2/4 plasmid as a template, the coding region fragment of the LcCHS2/4 gene was amplified via PCR. This segment was fused with the GFP coding sequence and ligated into the pCAM1302 vector, which had been linearized by double digestion with NcoI and SpeI, thereby constructing two fusion expression vectors, 35S::LcCHS2::GFP and 35S::LcCHS4::GFP, respectively, both driven by the 35S promoter (the recombinant plasmids were named pCAM1302-LcCHS2/4-GFP-His). The recombinant product was transformed into DH5α E. coli competent cells. Positive clones were screened via PCR analysis of bacterial cultures and validated by sequencing. The verified plasmid was extracted and chemically transformed into Agrobacterium tumefaciens GV3101 competent cells. PCR identification confirmed the positive engineered strain for subsequent experiments.

### 4.11. Subcellular Localization of Tobacco

The cultured Agrobacterium suspension containing the target gene was centrifuged at 5000× *g* for 10 min, and the bacterial cells were collected. The cells were resuspended in buffer (97 mL sterile distilled water + 1 mL 0.5 M MES (pH 5.6) + 1 mL 1 M MgCl_2_ + 200 μL 0.1 M As) to an OD of 0.2. Using a 1 mL disposable syringe, inject the resuspended bacterial suspension onto the abaxial surface of *Nicotiana benthamiana* leaves approximately 25 days post-transplanting. An empty vector was used as the control. After incubating in darkness at 25 °C for 12 h, transfer the plants to normal light conditions for 48 h of cultivation. A 0.2 mm × 0.2 mm microscopic sample was excised from the inoculated leaf using a scalpel and observed under a laser confocal microscope (NIS-Elements 6.0) for imaging.

### 4.12. Cis Elements Analysis in the Promoter of LcCHS Family Gene

The 2000 bp sequences upstream of the start codon of the litchi *CHS* gene family members were downloaded as the promoter region. Cis elements 2000 bp upstream of the litchi *LcCHS1-LcCHS6* gene promoters were extracted and profiled by PlantCARE online software (https://bioinformatics.psb.ugent.be/webtools/plantcare/html/, accessed on 28 April 2025) [59], and then were visualized using TBtools [53]. 

## 5. Conclusions

This study identified six *CHS* genes in litchi, designated *LcCHS1–LcCHS6*. These genes showed conserved gene structures, motifs, and predicted protein structures, suggesting that the CHS family is relatively conserved in litchi. Phylogenetic and collinearity analyses indicated that *LcCHS* genes are closely related to homologs from Sapindaceae species, especially longan, although further evolutionary analyses are needed. Expression analysis revealed tissue-specific and cultivar- or stage-dependent patterns during fruit development. Gene expression of *LcCHS* members highly correlate with flavonoid content. Promoter and correlation analyses suggested that *LcCHS* genes may be related to developmental, hormonal, stress-response, and flavonoid-associated regulatory pathways. Overall, this study provides candidate genes and potential molecular resources for future functional studies and breeding programs aimed at improving litchi fruit color, nutritional quality, and commercial value.

## Data Availability

The original contributions presented in this study are included in the article/Supplementary Material. Further inquiries can be directed to the corresponding authors.

## Supplementary Materials

The following supporting information can be downloaded at https://www.mdpi.com/article/10.3390/ijms27146152/s1.

## Abbreviations

The following abbreviations are used in this manuscript:

FZX	Feizixiao
ZNX	Ziniangxi
JGHN	Jinggang Hongnuo
