# Supplementary material for: Identification and Expression Profile of the Chalcone Synthase (CHS) Gene Family in Litchi
Source: Int J Mol Sci. 2026 Jul 9;27(14):6152. doi: 10.3390/ijms27146152 (PMC13410880; doi:10.3390/ijms27146152)
Supplement: Supplementary file 1 [file ijms-27-06152-s001.zip › ijms-4353089-Figure S1.pdf]

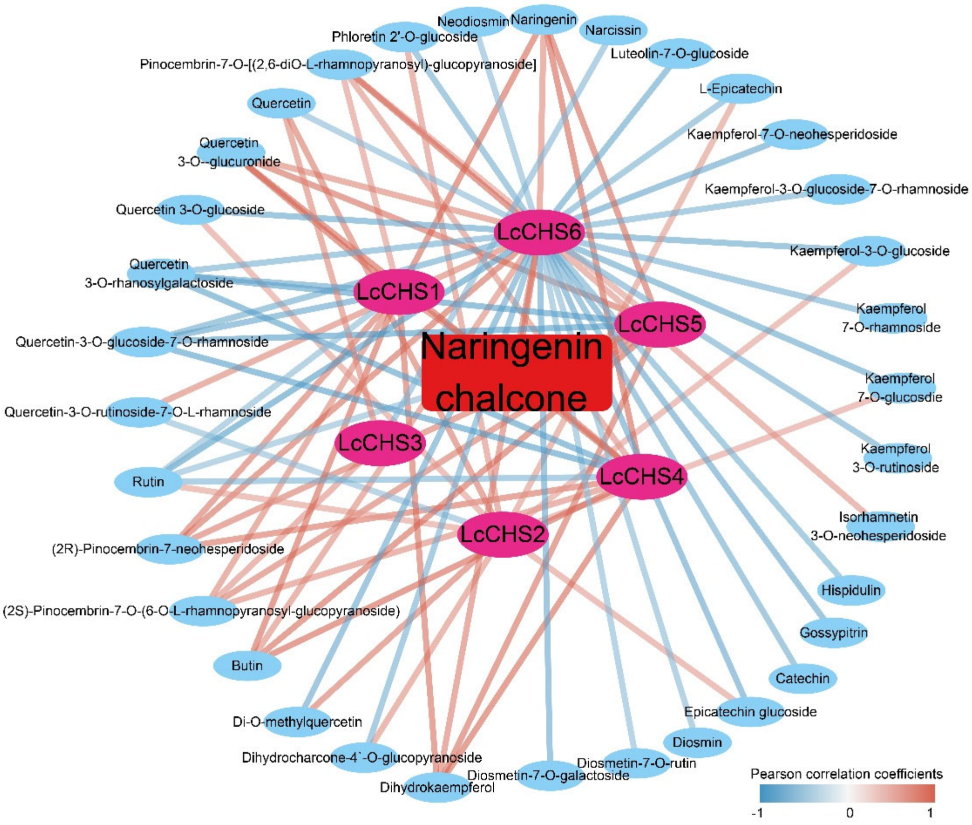

Figure S1. Correlation network between the gene expression of LcCHSs and flavonoids content in 'Guiwei' litchi.
